# Nanoplastics Penetrate Human Bronchial Smooth Muscle and Small Airway Epithelial Cells and Affect Mitochondrial Metabolism

**DOI:** 10.3390/ijms25094724

**Published:** 2024-04-26

**Authors:** Ewa Winiarska, Monika Chaszczewska-Markowska, Daniel Ghete, Marek Jutel, Magdalena Zemelka-Wiacek

**Affiliations:** 1 Department of Clinical Immunology, Wroclaw Medical University, 51-616 Wroclaw, Poland; ewa.popowicz@student.umw.edu.pl (E.W.);; 2 Laboratory of Clinical Immunogenetics and Pharmacogenetics, Hirszfeld Institute of Immunology and Experimental Therapy, Polish Academy of Sciences, 53-114 Wroclaw, Poland; 3 Bioscience Technology Facility, Department of Biology, University of York, York YO10 5DD, UK; 4 ALL-MED Medical Research Institute, 53-201 Wroclaw, Poland

**Keywords:** nanoplastic, polystyrene particles, 3D holotomography, holotomography, bronchial smooth muscle cells (BSMC), small airway epithelial cells (SAEC), mitochondrial respiration, glycolysis, oxygen consumption rate (OCR), extracellular acidification rate (ECAR)

## Abstract

Micro- and nanoplastic particles, including common forms like polyethylene and polystyrene, have been identified as relevant pollutants, potentially causing health problems in living organisms. The mechanisms at the cellular level largely remain to be elucidated. This study aims to visualize nanoplastics in bronchial smooth muscle (BSMC) and small airway epithelial cells (SAEC), and to assess the impact on mitochondrial metabolism. Healthy and asthmatic human BSMC and SAEC in vitro cultures were stimulated with polystyrene nanoplastics (PS-NPs) of 25 or 50 nm size, for 1 or 24 h. Live cell, label-free imaging by holotomography microscopy and mitochondrial respiration and glycolysis assessment were performed. Furthermore, 25 and 50 nm NPs were shown to penetrate SAEC, along with healthy and diseased BSMC, and they impaired bioenergetics and induce mitochondrial dysfunction compared to cells not treated with NPs, including changes in oxygen consumption rate and extracellular acidification rate. NPs pose a serious threat to human health by penetrating airway tissues and cells, and affecting both oxidative and glycolytic metabolism.

## 1. Introduction

Microplastic particles (MPs), especially with a size smaller than 1 µm, called nanoplastics (NPs), have emerged as a new type of pollution and they can be easily absorbed by organisms [1,2,3]. There are reports that MPs and NPs can cause allergic reactions, gut barrier dysfunction, gastrointestinal and lung cancers, leukemias, or neurotoxic effects in humans [4,5,6,7,8,9,10]. There is a dramatically increasing number of reports on the presence of NPs in atmospheric air samples, as well as in food and water, indicating a potential risk of these particles crossing the mucosal barrier [11,12,13,14]. The most commonly encountered forms of particles are polyethylene, polypropylene, and polystyrene (PS), which, due to their extremely small sizes (Φ < 100 nm), have a high affinity for tissues and a great adsorption capacity [15,16].

However, the exact mechanisms of how plastics react and whether MPs or NPs can trigger reactions in living organisms remain uncertain. The literature contains numerous reports on the in vitro impact of MPs and NPs on the cell’s viability, apoptosis, oxidation stress, or inflammation [17,18]. However, there is a lack of literature regarding both mitochondrial and glycolytic activity after exposure to nanoplastics.

To elucidate the interaction between NPs and human respiratory health, this study focuses on two cell types: bronchial smooth muscle cells (BSMC) from healthy and asthmatic patients and small airway epithelial cells (SAEC). These cells are among the first to encounter inhaled particulates, including MPs and NPs, due to their direct exposure to the airway environment. BSMCs play a key role in regulating airway tone and responsiveness, which are often altered in asthma, a condition characterized by chronic inflammation and hyperresponsiveness of the airways [19,20]. SAECs form the lining of the airways and serve as a barrier to inhaled particles while participating in innate immune responses [21]. The choice of these cells is strategic; they provide a relevant model to study the initial interaction and potential impact of exposure to NPs on the respiratory system’s health and function. Understanding how NPs interact with these cells can shed light on the mechanisms underlying their potential to exacerbate respiratory conditions, such as asthma, and contribute to the development of respiratory diseases through their effects on cell viability, inflammation, and metabolic dysregulation. This focus aligns with growing concerns over the inhalation of plastic particles and their ability to penetrate deeper into the respiratory tract.

Cell processes, including activation, proliferation, and memory development, are driven by metabolic reprogramming. The pivotal roles of mitochondrial respiration and glycolysis are essential for supporting cellular functions [22]. Total intracellular adenosine triphosphate (ATP) is mainly generated by glycolysis and mitochondrial oxidative phosphorylation. Real-time analysis of these metabolic pathways in living cells offers functional kinetic measurements of cellular bioenergetics [23]. The Seahorse Cell MitoStress Test is specifically designed for use with Seahorse XF Analyzers. This kit is instrumental in investigating mitochondrial dysfunction, functional differences among cell types, drug candidates, and genetic or biochemical interventions. Simultaneous measurement of dioxygen (O_2_) and pH in real-time determines mitochondrial respiration (oxidative phosphorylation) via the oxygen consumption rate (OCR), the extracellular acidification rate (ECAR), and the total proton efflux rate (PER) [24]. OCR values are measured to determine the mitochondrial activity for each part of the assay (three measurements/part). Regents are added to determine the parameters of mitochondrial activity. (a) Basal respiration is the difference between baseline OCR and non-mitochondrial OCR. Oligomycin, a specific inhibitor of the ATPase complex V, is added to determine the portion of basal respiration that is ATP-linked respiration and proton-leak respiration. (b) Carbonyl cyanide-p-trifluoromethoxyphenyl-hydrazon (FCCP), a protonophore, is added to collapse the inner membrane gradient, driving the mitochondria to respire at its maximal rate. This determines maximal respiratory capacity. (c) Antimycin A and rotenone are complex III and I inhibitors which stop mitochondrial respiration to determine non-mitochondrial respiration. Reserve capacity is the difference between basal respiration and maximal respiratory capacity.

Live cell, label-free, real-time imaging provides essential information to the investigation of cell biology and related pathophysiology. The holotomography (HT) technology offers non-invasive, three-dimensional quantitative imaging capabilities that allow for the examination and analysis of cells and tissues without requiring any form of sample preparation such as fixation or staining [25,26]. Conventional imaging techniques used to visualize unstained biological specimens, such as phase contrast or differential interference contrast microscopy, are constrained by their ability to provide only two-dimensional representations, despite biological samples being inherently three-dimensional [27]. HT technology provided by Tomocube offers a non-invasive alternative that uses transmitted light to measure the intrinsic properties of the cells and images them in a label-free mode. It utilizes the refractive index (RI)—a fundamental optical characteristic that defines how light is changed when passing through a material—for the visualization of live cells and tissues. As light traverses through a sample, the various constituents scatter light differently based on their refractive index. By rotating the imaging beam around the sample in a full circle, a series of holograms are collected from multiple perspectives [28]. These collected two-dimensional (2D) holographic images, taken from different angles of illumination, are then compiled to construct a 3D RI tomogram, offering a more comprehensive view of the sample’s internal structure [29]. HT provides structural and biochemical information about the cell, including dry mass, morphology, and dynamics of the cellular membrane, and enables the visualization of the dynamic processes and mechanisms within living cells and their subcellular components.

This study aimed to do the following: (a) Visualize and confirm the entry of PS-NPs into BSMC and SAEC cells in real-life settings using the HT technology. Given that BSMC and SAEC typically do not phagocyte, it is crucial to confirm the cells’ internalization of plastic. (b) To assess that PS-NPs with a diameter of 25 and 50 nm influence the mitochondrial metabolisms in BSMC and SAEC cells in in vitro cultures.

## 2. Results

**Scheme 1:**
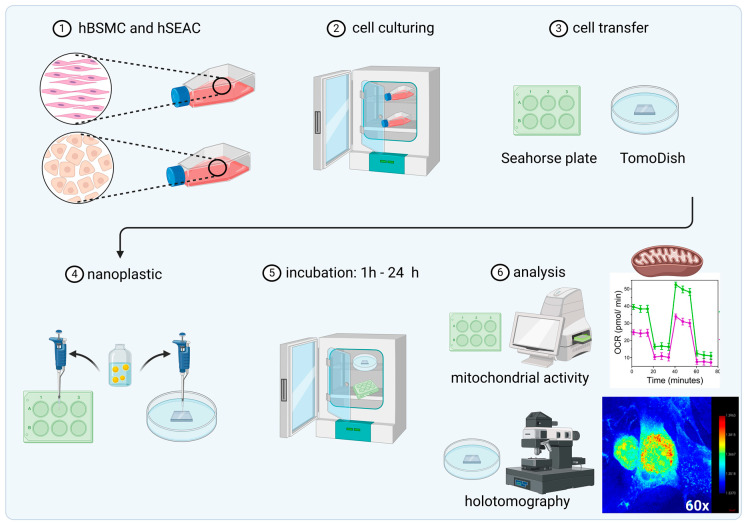
Experiment layout. Abbreviations: BSMC = human bronchial smooth muscle cells; SAEC = human small airway epithelial cells.

**Scheme 2:**
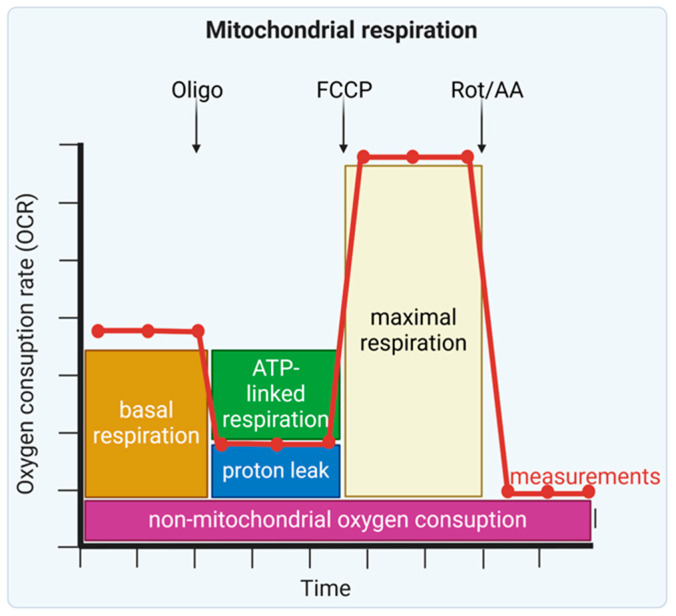
The Seahorse XF Cell MitoStress Test profile illustrates key parameters of mitochondrial function.

The comprehensive structure of the experiment is depicted in Scheme 1 and Scheme 2.

### 2.1. Visualization of NPs

**Figure 1:**
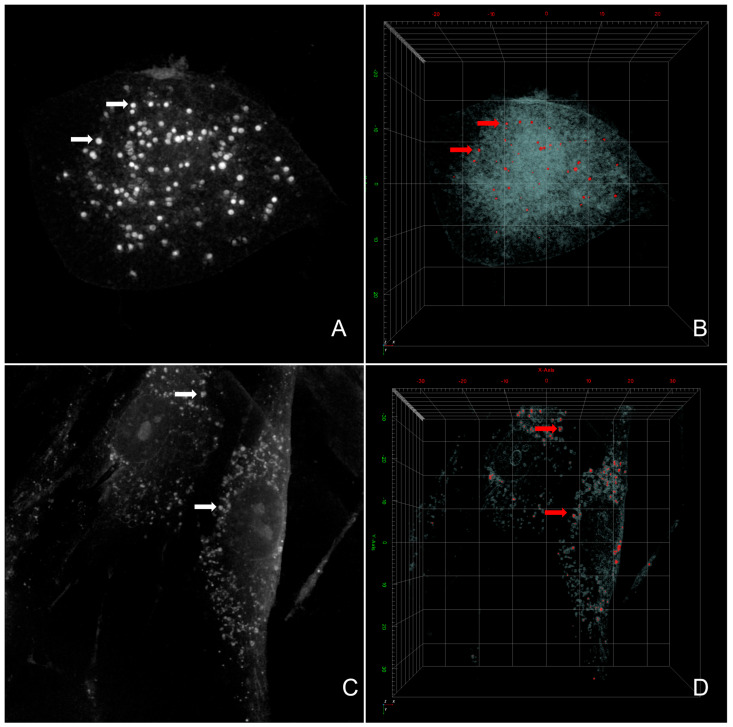
The holotomography images display cells with nanoplastics (NPs) inside, magnification 58.3, numerical-aperture 1.20. Images (**A**) and (**C**) represent maximum intensity projections (MIP) of cells containing NPs, shown by white arrows pointing at white dots in the cells, human small airway epithelial cells (SAECs) and healthy human bronchial smooth muscle cells (BSMCs), respectively. Images (**B**) and (**D**) show the 3D visualization of the same data, employing pseudo-coloring based on a ranges refractive index (RI) in SAECs and BSMCs, respectively. The objects (arrows and dots) appearing in red in images (**B**,**D**), denoting an RI greater than 1.40, correspond to the white objects identified in images (**A**,**C**).

Healthy BSMCs and SAECs were seeded into the TomoDish at densities of 3500 cells/dish and 2500 cells/dish, respectively, in 5 mL of either smooth muscle cell growth basal medium (SMBM) or small airway epithelial cell growth medium (SAGM). Cells were incubated at 37 °C in a 5% carbon dioxide (CO_2_) atmosphere. After 24 h, 50 nm PS-NPs were added at a final concentration of 100 μg/mL of medium. Following 1 h of treatment, the cells were analyzed using a holotomography microscope. NPs were visualized using HT, as their RI was higher than the RI of any organelles in the cells. The RI of NPs was above 1.4 (Figure 1).

Acknowledging the potential for nanoparticle or medium contamination, we have conducted experiments that evidenced the absence of antimicrobials and surfactants originating from the medium or nanoparticle suspension [30] (Supplementary Materials, Figure S1).

### 2.2. Mitochondrial Metabolism

BSMCs and SAECs were seeded into an 8-well cartridge at densities of 6000 and 7000 cells per dish, respectively, using 180 μL of SMBM or SAGM. The cells were incubated at 37 °C in a 5% CO_2_ atmosphere. After 24 h, PS-NPs of 25 or 50 nm were added at a concentration of 100 μg/mL of medium for durations of 1 or 24 h. In the control wells, no NPs were added. The confluence of cells was 70%. Prior to testing, the medium was replaced with a supplemented Dulbecco’s Modified Eagle Medium (DMEM) pH 7.4. Each sample was replicated thrice (in each experiment). Two wells in each cartridge were filled only with medium for calibration purposes. The protocol was executed following the manufacturer’s recommendations. The results represent the difference between the test sample and the background (medium).

**Figure 2:**
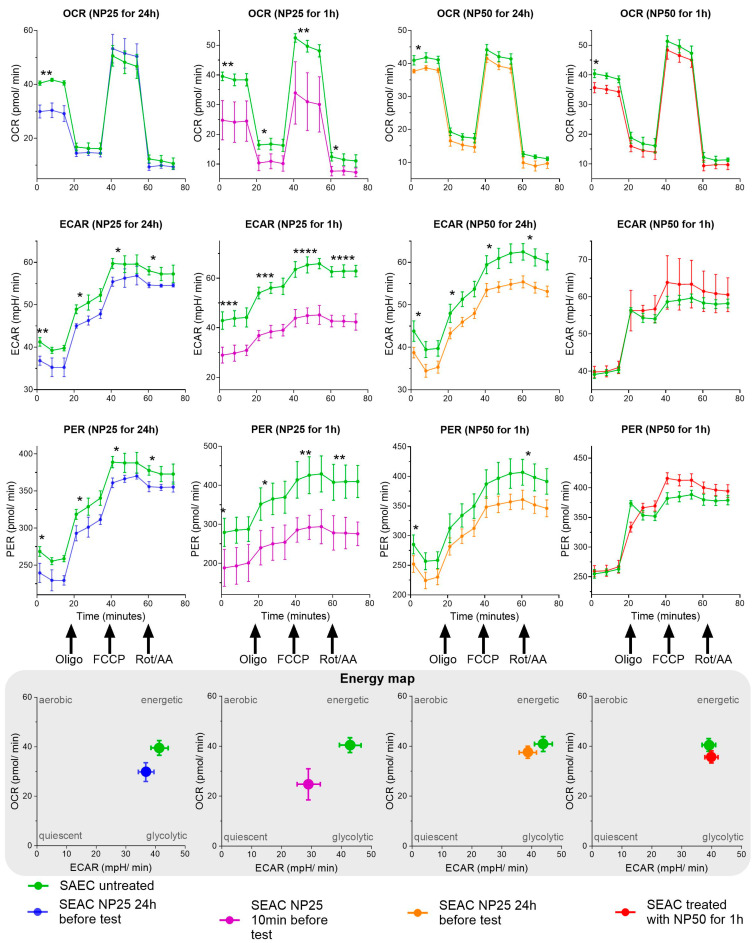
Human small airway epithelial cells (SAEC) have impaired bioenergetics and dysfunctional mitochondria following exposure to polystyrene nanoplastics (PS-NPs) of 25 nm and 50 nm in diameter for durations of 1 h and 24 h, compared to SAEC that were not exposed to NPs. Measurements of oxygen consumption rate (OCR), extracellular acidification rate (ECAR), and total proton efflux rate (PER) were conducted under basal conditions, and in response to the following mitochondria inhibitors: oligomycin (Oligo), cyanide-4-(trifluoromethoxy)phenylhydrazone (FCCP), and rotenone plus antimycin A (Rot/AA). Data represents mean values with standard deviation (SD), * *p* < 0.05, ** *p* < 0.01; *** *p* < 0.001, and **** *p* < 0.0001.

SAEC exposed to 25 or 50 nm PS-NPs for durations of 1 or 24 h exhibited a statistically significant reduction in OCR compared to the control group that was not treated with NPs (Figure 2). For the short, 1 h exposure duration, the reductions in OCR were as follows: 25 nm PS-NPs (*p* < 0.01) and 50 nm PS-NPs (*p* < 0.05), both observed at measurement number one. Moreover, during this exposure period, SAECs exposed to 25 nm PS-NPs demonstrated a statistically significant decrease in both ECAR (*p* < 0.001) and PER (*p* < 0.05). However, the response to 50 nm PS-NPs for 1 h did not show a statistically significant change in ECAR and PER, which were marked as not significant (ns). In the case of long, 24 h exposure durations, the situation further evolves. The reductions in OCR for SAECs were detailed as follows: 25 nm PS-NPs (*p* < 0.01) and 50 nm PS-NPs (*p* < 0.05). This long exposure also led to a statistically significant decrease in both ECAR and PER for cells exposed to 25 or 50 nm PS-NPs. Specifically, the statistical significances were as follows: for ECAR, 25 nm PS-NPs (*p* < 0.01) and 50 nm PS-NPs (*p* < 0.05); for PER, both 25 nm and 50 nm PS-NPs exhibited similar trends with statistical significances marked as (*p* < 0.05). These findings were consistently observed at subsequent measurements when adding different inhibitors, highlighting the impact of nanoparticle exposure on SAEC.

**Figure 3:**
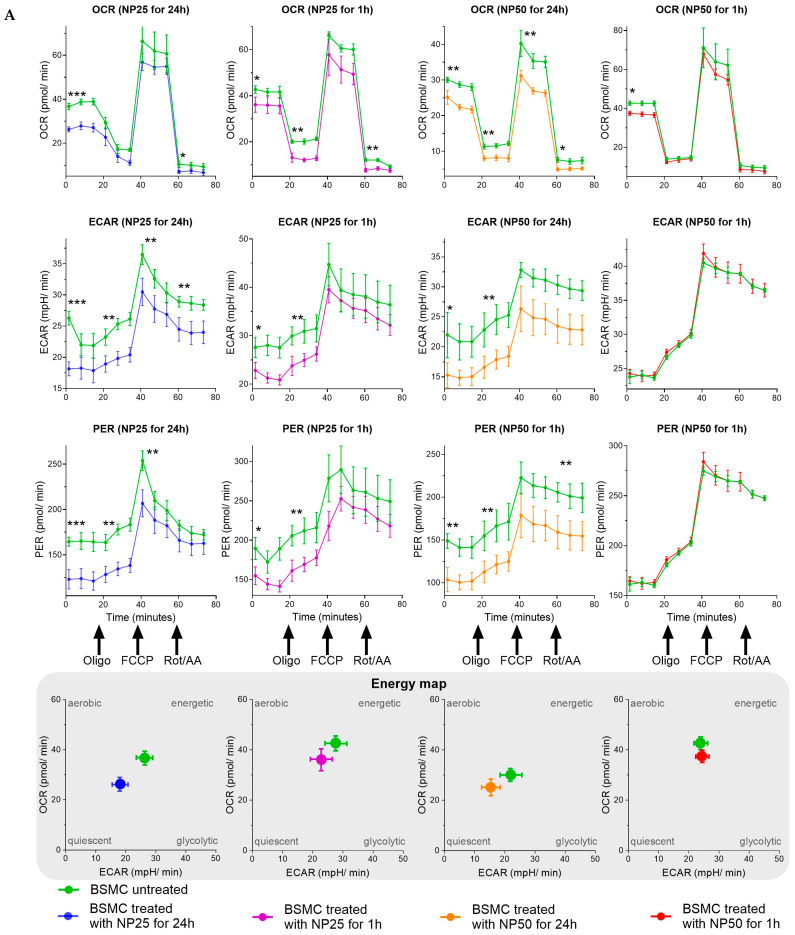

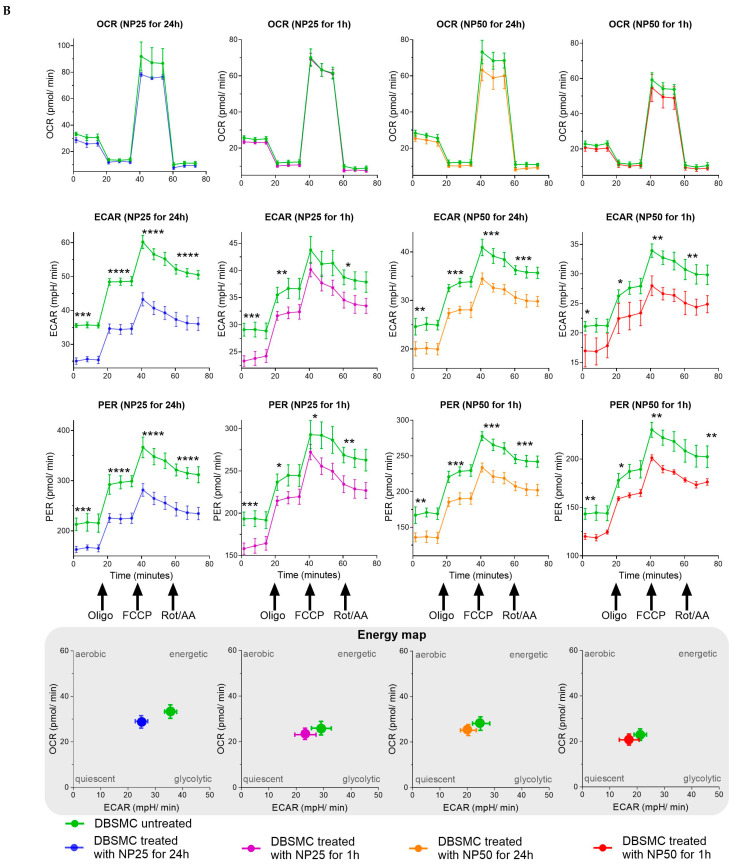
The impact of polystyrene nanoplastics (PS-NPs) of 25 nm and 50 nm in diameter on the mitochondrial metabolism (**A**) in human healthy bronchial smooth muscle cells (BSMC) and (**B**) asthmatic (diseased) bronchial smooth muscle cells (DBSMC) following exposure periods of 1 h and 24 h, in comparison to BSMC or DBSMC that were not treated with NPs. The assessment included measurements of oxygen consumption rate (OCR), extracellular acidification rate (ECAR), and total proton efflux rate (PER) under basal conditions, as well as in response to the following mitochondrial inhibitors: oligomycin (Oligo), cyanide-4-(trifluoromethoxy)phenylhydrazone (FCCP), and a combination of rotenone and antimycin A (Rot/AA). The data are presented as mean values accompanied by standard deviations (SD), * *p* < 0.05, ** *p* < 0.01; *** *p* < 0.001, and **** *p* < 0.0001.

BSMC and DBSMC were exposed to 25 or 50 nm PS-NPs for 1 h and 24 h, respectively. These exposures led to variations in metabolic functions compared to the control group that was not treated with NPs, as indicated by changes in OCR, ECAR, and PER (Figure 3A,B). For the short-duration exposure (1 h), BSMC exposed to 25 nm and 50 nm PS-NPs for 1 h exhibited a statistically significant reduction in OCR (25 nm: *p* < 0.05, 50 nm: *p* < 0.05), as observed at measurement number one. However, the impact on ECAR and PER was variable; exposure to 25 nm PS-NPs significantly decreased ECAR (*p* < 0.05) and PER (*p* < 0.05), while exposure to 50 nm PS-NPs did not produce significant changes in ECAR (ns) and resulted in non-significant changes in PER (ns). In contrast, DBSMC displayed distinct responses; both 25 nm and 50 nm PS-NP exposures resulted in significant alterations in ECAR (25 nm: *p* < 0.001, 50 nm: 0.05) and PER (25 nm: *p* < 0.001, 50 nm: *p* < 0.01), and no significant changes in OCR were noticed, highlighting a differential sensitivity to nanoparticle size and exposure duration. For the long-duration exposure (24 h), BSMC treated with 25 nm and 50 nm PS-NPs showed more pronounced decreases in OCR (25 nm: *p* < 0.001, 50 nm: *p* < 0.01). The long-duration exposure also significantly reduced ECAR (25 nm: *p* < 0.001, 50 nm: *p* < 0.05) and PER (25 nm: *p* < 0.001, 50 nm: *p* < 0.01) compared to untreated control groups. Similar trends were observed in DBSMC, where 24 h exposure to 25 nm and 50 nm PS-NPs markedly affected ECAR (25 nm: *p* < 0.001, 50 nm: *p* < 0.01) and PER (25 nm: *p* < 0.001, 50 nm: *p* < 0.01), and there were no significant changes in OCR.

## 3. Discussion

The field of in vitro studies on MPs and NPs has extensively explored their effects across a variety of cell types, including epithelial cells, embryonic stem cells, carcinoma cell lines, hepatocarcinoma cells, gastric adenocarcinoma cells, colon cells, fibroblast cells, monocytes, leukocytes, and others [31,32,33]. These investigations predominantly focus on cellular uptake, viability, apoptosis, oxidative stress, and the release of pro-inflammatory interleukins, employing advanced techniques such as confocal microscopy to visualize plastics stained with fluorochromes. This body of research lays a foundational understanding of the cellular interactions with MPs and NPs, highlighting the diverse cellular responses to plastic exposure [34,35,36].

Holotomography represents a significant innovation in the field of live cell imaging, primarily due to its unique approach to capturing high-resolution, 3D images of living cells without the need for labeling or staining. This method is designed to provide meaningful insight into changes in physical parameters at the single-cell level. Traditional imaging techniques often require fluorescent labels or dyes to visualize cellular components, which can be toxic and potentially alter cellular behavior. HT, by contrast, does not require any labeling, thereby allowing the observation of cells in their natural, unaltered state. RI is a very specific and sensitive parameter. That is why when we have points with high density, as in the case of NPs, we can see it very clearly, and it is reflected in very high RI. There is no possibility to have artifacts because of very precision measurements. Here, we demonstrate for the first time that PS-NPs are visualized in live cell imaging in human airway cells—SAEC and BSMC [37]. Furthermore, HT is a quantitative imaging method that allows us to measure the dry mass of lipids and proteins inside the cells in 3D. Different organelles in mammalian cells can be visualized based on RI [38]. The objects that have the highest density and highest RI inside the cells are the lipid droplets [39]. The RI ranges of mammalian cells were shown to be between 1.337 and 1.4, with the highest RI being lipid droplets inside the cells [40]. As the NPs are uptaken by the cells, they aggregate in spherical objects that have an RI higher than 1.4, which indicates a denser material than lipids, which are usually in lipid droplets at an RI below 1.4. This is similar to the normal uptake of any type of nanomaterial that has a higher RI than what can be found normally in cells [41,42]. With HT, we can confirm the uptake of NPs and visualize them in 3D inside of the cells.

Furthermore, our research utilizes Seahorse XF Analyzers to delve into the metabolic activities of cells in real-time. The OCR serves as an indicator of mitochondrial respiration, the primary process responsible for ATP (adenosine triphosphate) production in aerobic organisms, while ECAR is primarily utilized as an indicator of glycolysis, a process that metabolizes glucose without oxygen to produce ATP [43]. Such measurements are crucial for understanding the cellular responses and understanding that the metabolic phenotype (the balance between oxidative phosphorylation indicated by OCR, and glycolysis changes indicated by ECAR) can indicate how cells adapt their metabolic pathways in response to stress, drug treatment, or changes in their environment [44,45]. 

Our findings suggest that exposure of SAEC, BSMC, or DBSMC to NPs decreases both oxidative phosphorylation and glycolysis across all tested groups, regardless of the size of the plastic particles or the duration of exposure. Notably, asthmatic BSMC cells exhibit higher sensitivity to 50 nm NPs compared to their non-asthmatic counterparts or SAEC, underscoring the increased vulnerability of diseased cells to nanoplastic exposure. Additionally, it is notable that the more significant changes occur in ECAR in all tested groups, which specifically measures the proton accumulation in the medium surrounding cells, which occurs as a byproduct of lactic acid production during glycolysis.

Based on the measurement, the energy map differentiates cells into four categories based on their metabolic states. (a) Aerobic: cells predominantly utilize aerobic respiration for energy production. This indicates a high reliance on oxygen to metabolize glucose through the tricarboxylic acid cycle and oxidative phosphorylation, leading to efficient ATP generation with minimal lactate production. (b) Energetic: cells exhibit high levels of both aerobic respiration and glycolysis. This dual approach suggests that these cells can rapidly adapt to changes in their environment by efficiently utilizing both oxygen-dependent and -independent pathways for energy production. (c) Quiescent: cells show low metabolic activity, indicating minimal engagement in both glycolysis and aerobic respiration. This state is often associated with cells in a resting phase or with limited energy requirements, conserving resources until activation is necessary. (d) Glycolytic: cells rely on glycolysis for energy production, to generate ATP quickly without the need for oxygen. This state is characteristic of rapidly proliferating cells and those in hypoxic conditions. Our study demonstrates the metabolic shifts induced by NP exposure, notably transitioning from an energetic phenotype to glycolytic/quiescent states, observed across all cell types and experimental conditions (25 or 50 nm NPs, for 1 or 24 h). Such shifts suggest a fundamental alteration in cellular energy production mechanisms in response to nanoplastic exposure. However, the study has limitations, notably the inability to compare the responses of SAEC with BSMCs, including distinctions between healthy and asthmatic BSMCs, due to the experimental design.

Our observations are corroborated by previous studies that have documented the detrimental effects of NPs on cellular mitochondria. For instance, exposure to PS-NH_2_ (60 nm) particles has been shown to induce mitochondrial damage in macrophages, leading to a vacuolar appearance that is indicative of mitochondrial origin [46]. The primary mechanism of toxicity is believed to be the elevation of reactive oxygen species (ROS) from oxidative stress, which compromises mitochondrial membrane potential and functionality [47,48]. Furthermore, recent research has highlighted the direct link between microplastic exposure and mitochondrial damage, emphasizing the consequential release of mitochondrial DNA into the cytoplasm as a marker of mitochondrial dysfunction [49]. In another study, the exposure of a monoculture of human brain vascular pericytes to polyethylene terephthalate particles in vitro did not elicit oxidative stress, but augmented various aspects of mitochondrial respiration [50]. However, different research showed that NPs did not induce the generation of ROS, as supported by the observation that there were no changes in either the OCR or ECAR during mitochondrial respiration in rainbow trout head kidney macrophages (RT-HKM) [51].

Despite these advancements, there remains a need for further research to broaden our understanding of NP-induced mitochondrial metabolism impairment across different cell types, plastic types, concentrations, and exposure durations. The emerging evidence underscores the critical impact of NPs on mitochondrial function, signaling a pivotal area for future investigations to elucidate the full spectrum of nanoplastic toxicity.

## 4. Materials and Methods

### 4.1. Cells

Healthy and diseased asthmatic human bronchial smooth muscle cells (BSMC) and human small airway epithelial cells (SAEC) were obtained from Lonza Group Ltd. Visp, Switzerland as the following commercially available products: BSMC (# CC-2576), diseased BSMC (# 00194850), and SAEC (# CC-2547).

### 4.2. Cell Cultures

Healthy and asthmatic BSMCs were stored in liquid nitrogen at an initial concentration of 500,000 cells per vial. These cells were cultured in a medium and passaged after reaching 80% confluency, adhering to the manufacturer’s protocol. Cells were cultured in flasks ranging from 25 to 175 cm^2^, maintained at 37 °C in a 5% CO_2_ atmosphere, in an SMBM supplemented with insulin, human fibroblast growth factor—basic, gentamicin-amphotericin, human epidermal growth factor, and fetal bovine serum (FBS) as per the Lonza SMBM kit CC-3182. Passages P3 to P5 were selected for this study. For mitochondrial analysis, cells were plated on Seahorse assay cartridges at a density of 6000 cells per well 24 h prior to each experiment. For holotomography experiments, cells were plated on TomoDish (Tomocube Inc., Daejeon, Republic of Korea) at a density of 3500 cells per dish 24 h before the experiment.

SAECs were stored in liquid nitrogen at an initial concentration of 500,000 cells per vial. These cells were cultured in a medium and passaged after reaching 80% confluency, adhering to the manufacturer’s protocol. Cells were cultured in flasks ranging from 25 to 175 cm^2^, maintained at 37 °C in a 5% CO_2_ atmosphere, in a SAGM enriched with bovine pituitary extract, insulin, hydrocortisone, gentamicin-amphotericin, retinoic acid, transferrin, triiodothyronine, epinephrine, and human epidermal growth factor. For this experiment, passages P4 to P7 were utilized. The mitochondrial analysis required seeding cells on Seahorse assay cartridges at a density of 7000 cells per well 24 h in advance of the experiment. For HT, cells were seeded on TomoDish (Tomocube Inc., Republic of Korea) at a density of 2500 cells per dish.

### 4.3. Nanoplastics

Polystyrene nanoparticles (PS-NPs) with diameters of 25 nm and 50 nm, featuring plain surfaces, and primary concentrations of 10 mg/mL were supplied by micromod Partikeltechnologie GmbH, Rostock, Germany. The manufacturer washed and then suspended the particles in deionized water, which was produced through reverse osmosis; no surfactants were added. NPs were added to cultured cells at different times and concentrations, listed in the results section.

### 4.4. Holotomography Microscope

The HT was performed using a commercial optical diffraction tomography (ODT) microscope (HT-2H; Tomocube Inc., Republic of Korea). This microscope is based on Mach–Zehnder interferometry and equipped with a digital micromirror device (DMD). A coherent monochromatic laser (532 nm) was divided into the following two light paths: a reference and a sample beam. A 3D RI tomogram was reconstructed from multiple 2D holographic images acquired from 49 illumination conditions. A normal incidence and 48 azimuthally symmetric directions with a polar angle (64.5°) were used. The DMD was used to control the angle of an illumination beam projected onto the sample. The diffracted beams from the sample were collected using a high numerical aperture (NA) objective lens (NA = 1.2, UPLSAP 60XW, Olympus, Tokyo, Japan). The off-axis hologram was recorded using a complementary sCMOS image sensor (Blackfly S BFS-U3-28S5M, FLIR Systems Inc., Wilsonville, OR, USA). The data were imaged and visualized using commercial software (TomoStudio HT-2H Gen3—3.3.9, Tomocube Inc., Republic of Korea).

### 4.5. Mitochondrial Analysis

The Seahorse XF HS Mini Analyzer (Agilent Technologies, Santa Clara, CA, USA), along with the XFp Cell MitoStress Test Kit and XFp FluxPak (8-well cartridge designed for adherent cells), were utilized in the experiments. Cells were transferred onto Seahorse assay plates, and in some groups they were treated with PS-NP of either 25 nm or 50 nm in diameter, 1 h or 24 h prior to the assessment. All procedures adhered to the manufacturer’s standard protocols. The medium was replaced with DMEM, pH 7.4, which was supplemented with pyruvate, glucose, and glutamine solutions, as provided by Agilent Technologies. OCR was measured in real-time, under basal conditions, and in response to the following mitochondria inhibitors: oligomycin (Oligo)—1.5 μM, carbonyl cyanide-p-trifluoromethoxyphenyl-hydrazon (FCCP)—1.5 μM, and rotenone plus antimycin A (Rot/AA)—0.5 μM.

The experimental setup utilized an 8-well cartridge, incorporating two background controls and two sets of triplicates for the control and treatment groups. This accounts for the multiple graphs presented. Across these setups, we consistently compared the control group to a single treatment condition.

### 4.6. Statistical Analysis

The groups were compared using the t-test. Values were considered significant for all tests with *p* < 0.05. Statistics were calculated and graphs were drawn using GraphPad Software 10.2.2, Boston, MA, USA, www.graphpad.com (accessed on 23 April 2024).

## 5. Conclusions

This study shows for the first time in life-cell imaging (holotomography) the capacity of NPs to infiltrate the human airway tissues and enter the following structural cells: SAECs and healthy and diseased BSMCs. We postulate that the main nanoplastics mechanism affecting human health is related to oxidative and glycolytic metabolism impairment.

## Data Availability

All data are archived at the Department of Clinical Immunology, Wroclaw Medical University, Poland, and can be provided upon request by the corresponding author.

## Supplementary Materials

The following supporting information can be downloaded at: https://www.mdpi.com/article/10.3390/ijms25094724/s1.
