# Supplementary material for: Nanoplastics Penetrate Human Bronchial Smooth Muscle and Small Airway Epithelial Cells and Affect Mitochondrial Metabolism
Source: Int J Mol Sci. 2024 Apr 26;25(9):4724. doi: 10.3390/ijms25094724 (PMC11083782; doi:10.3390/ijms25094724)

Supplementary Materials

Nanoplastics Penetrate Human Bronchial Smooth Muscle and Small Airway Epithelial Cells and Affect Mitochondrial Metabolism

Ewa Winiarska, Monika Chaszczewska-Markowska, Daniel Ghete, Marek Jutel and Magdalena Zemelka-Wiacek

Figure S1. Holotomography examples of BSMCs (top 2 rows of images) and SAEC (bottom 2 rows of images) imaged upon uptake of nanoplastics. Images from each row are the same image displayed differently. The images on the left are inverse greyscale images where the low refracting index (RI) is white, and the high RI is black. The images in the middle are in greyscale where low RI is black and high RI is white. The images on the right JET coloring where low RI is blue and high RI is red. The highest RI measured in these images is above 1.40. The method is designed for single-cell analysis with high resolution, allowing for the detection of any artifacts, including bacteria. The absence of artifacts originating from the medium or nanoparticle suspension is clearly evident.

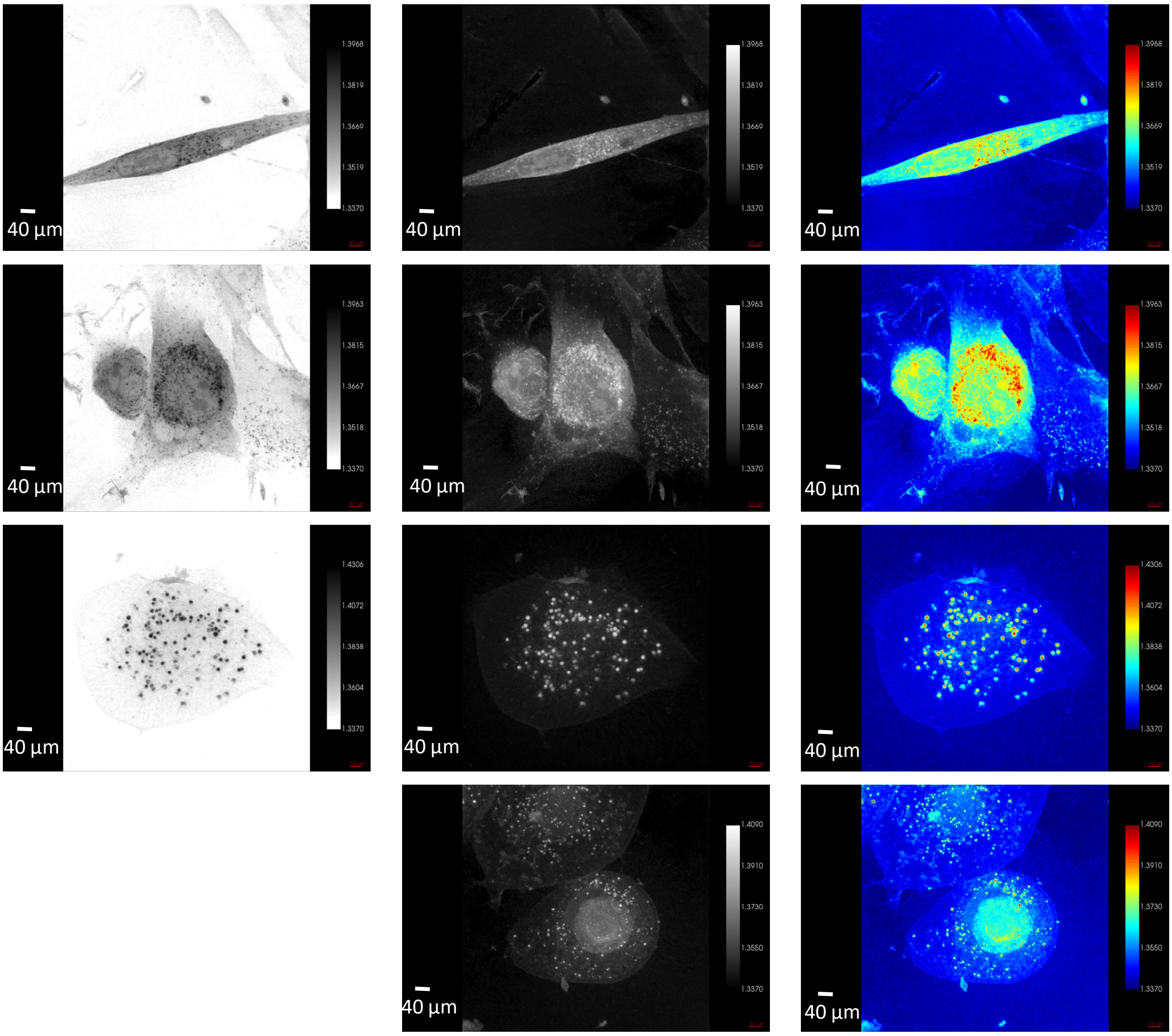

Supplement: Supplementary file 1 [file ijms-25-04724-s001.zip › ijms-2959657-supplementary.pdf]
